# Stromal interaction molecule 1/microtubule‐associated protein 1A/1B‐light chain 3B complex induces metastasis of hepatocellular carcinoma by promoting autophagy

**DOI:** 10.1002/mco2.482

**Published:** 2024-02-09

**Authors:** Jingchun Wang, Qichao Xie, Lei Wu, Yu Zhou, Yanquan Xu, Yu Chen, Jiangang Zhang, Ran Ren, Shiming Yang, Yongsheng Li, Huakan Zhao

**Affiliations:** ^1^ Department of Gastroenterology Second Affiliated Hospital Army Medical University Chongqing China; ^2^ Department of Oncology The Third Affiliated Hospital Chongqing Medical University Chongqing China; ^3^ Department of Medical Oncology Chongqing University Cancer Hospital Chongqing China; ^4^ Clinical Medicine Research Center Second Affiliated Hospital Army Medical University Chongqing China

**Keywords:** autophagy, epithelial–mesenchymal transition/EMT, hepatocellular carcinoma/HCC, microtubule‐associated protein 1A/1B‐light chain 3B/LC3B, stromal interaction molecule 1/STIM1

## Abstract

Metastasis is the leading cause of death in hepatocellular carcinoma (HCC) patients, and autophagy plays a crucial role in this process by orchestrating epithelial–mesenchymal transition (EMT). Stromal interaction molecule 1 (STIM1), a central regulator of store‐operated calcium entry (SOCE) in nonexcitable cells, is involved in the development and spread of HCC. However, the impact of STIM1 on autophagy regulation during HCC metastasis remains unclear. Here, we demonstrate that STIM1 is temporally regulated during autophagy‐induced EMT in HCC cells, and knocking out (KO) STIM1 significantly reduces both autophagy and EMT. Interestingly, STIM1 enhances autophagy through both SOCE‐dependent and independent pathways. Mechanistically, STIM1 directly interacts with microtubule‐associated protein 1A/1B‐light chain 3B (LC3B) to form a complex via the sterile‐α motif (SAM) domain, which promotes autophagosome formation. Furthermore, deletion of the SAM domain of STIM1 abolishes its binding with LC3B, leading to a decrease in autophagy and EMT in HCC cells. These findings unveil a novel mechanism by which the STIM1/LC3B complex mediates autophagy and EMT in HCC cells, highlighting a potential target for preventing HCC metastasis.

## Graphical abstract

The role of STIM1 in regulating autophagy during HCC metastasis remains unclear. In this study, the authors demonstrate that STIM1 enhances autophagy flux by directly binding with LC3B, independently of SOCE. Mechanistically, STIM1 interacts with LC3B through the SAM domain, promoting the formation of autophagosomes. These findings suggest a potential therapeutic approach for preventing HCC metastasis.

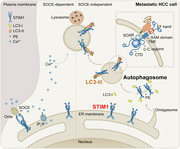

## 1 INTRODUCTION

Hepatocellular carcinoma (HCC) is the fifth most frequent malignancy and the third leading cause of cancer‐related deaths worldwide.^1^ Despite the application of multiple innovative therapeutic strategies, the outcome of HCC remains unfavorable, with an overall 5‐year survival rate of approximately 30% after resection.^2^ Metastasis is the primary cause of poor prognosis and high mortality in HCC, and it involves a complex series of invasion‐metastasis cascades.^3^ Epithelial–mesenchymal transition (EMT) is a fundamental cellular process in which cells lose their epithelial characteristics, such as high E‐cadherin expression, and acquire mesenchymal features, such as high N‐cadherin and Vimentin expression.^4^ EMT has been implicated in tumorigenesis and metastasis as it gives cancer cells enhanced mobility, invasion capabilities, resistance to apoptotic stimuli, and stem cell properties. Understanding the regulatory factors of EMT is crucial for the development of effective therapeutic interventions against metastasis.

Accumulating evidence suggests that autophagy plays a crucial role in EMT process, making it a potential target for preventing HCC metastasis.^5^, ^6^ Autophagy, an important self‐protective mechanism of cancer cells, regulates the orderly degradation and recycling of cellular components.^7^ This process involves sequestering waste products into autophagosomes, which are then degraded by lysosomes. The initiation of autophagy occurs in the endoplasmic reticulum (ER), where a double‐membrane autophagosome is formed for the degradation of sequestered materials.^7^, ^8^ The conversion from microtubule‐associated protein 1A/1B‐light chain 3B (LC3B‐I, unconjugated form) to LC3B‐II (conjugated form) is a prerequisite for the expansion of the phagophore and the formation of autophagosomes.^9^ Multiple inducers of EMT, such as hypoxia,^10^ hepatocyte growth factor,^11^ and transforming growth factor beta,^12^ have been shown to activate autophagy. Furthermore, studies have demonstrated that Unc‐51 like autophagy activating kinase 2 (ULK2), an important regulator of autophagy, plays a crucial role in the modulation of EMT in A549 cells.^13^ Therefore, it is essential to gain a deeper understanding of the regulatory mechanisms of autophagy in the metastasis of HCC.

Calcium ions (Ca^2+^) transient is pivotal for regulating cancer cell proliferation, apoptosis, and metastasis.^14^ In nonexcitable cells like cancer cells, the store‐operated Ca^2+^ entry (SOCE) serves as the primary pathway for extracellular Ca^2+^ influx.^15^ Stromal interaction molecule 1 (STIM1), a transmembrane Ca^2+^ sensor in the ER, plays a key role in maintaining calcium homeostasis by initiating the SOCE process after ER luminal Ca^2+^ store depletion.^16^, ^17^, ^18^ STIM1 consists of an ER luminal EF‐hand and a sterile‐α motif (SAM) that includes two N‐linked glycosylation sites. The C‐terminal of STIM1 is located in the cytosol and comprises three coiled‐coil regions, followed by a serine/proline‐rich and a lysine‐rich region.^19^ In previous studies, we discovered that STIM1 plays a role in stabilizing hypoxia‐inducible factor‐1 alpha (HIF‐1α) by activating Ca^2+^/calmodulin‐dependent protein kinase II (CaMKII). This activation process facilitates hypoxia‐driven hepatocarcinogenesis.^20^ Additionally, the STIM1‐snail family transcriptional repressor 1 (Snail1) negative feedback circuit alters the metabolism from aerobic glycolysis to fatty acid oxidation, thereby promoting invasion and metastasis of HCC.^21^

Although a previous study reported that oxidized low‐density lipoprotein (ox‐LDL) enhanced protective autophagy through the SOCE‐CaMKII‐mammalian target of rapamycin pathway in endothelial progenitor cells,^22^ the specific role of STIM1 in regulating autophagy in HCC cells has not been clarified. In this study, our objective is to investigate the role and mechanism of STIM1 in mediating autophagy in HCC cells. Our findings reveal a SOCE‐independent function of STIM1 in the autophagy process, providing a potential therapeutic alternative for metastatic HCC.

## 2 RESULTS

### 2.1 STIM1 is temporally regulated during autophagy‐triggered EMT in HCC cells

**FIGURE 1:**
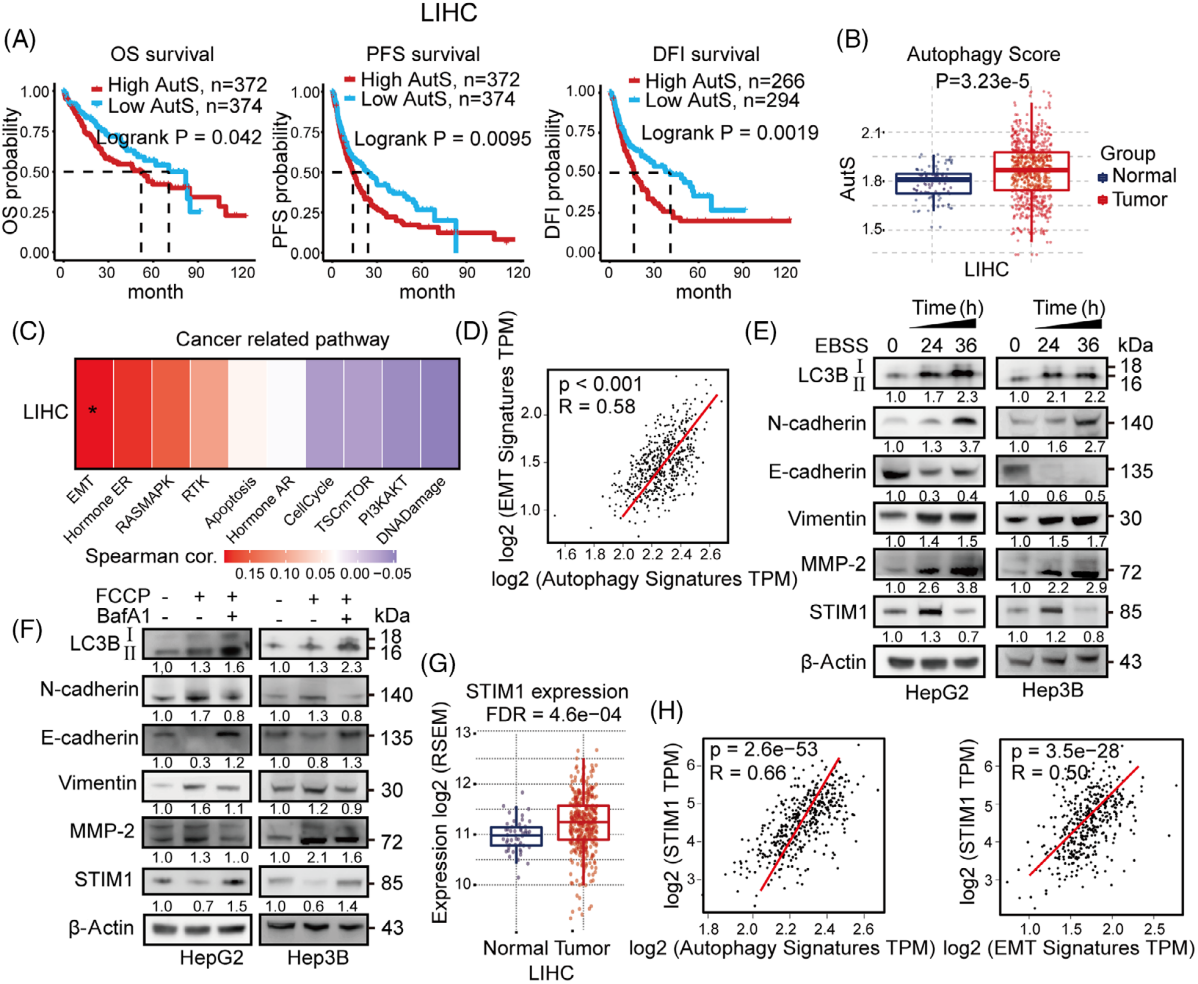
STIM1 is temporally regulated during autophagy‐induced EMT in HCC cells. (A) Comparison of overall survival (OS), progression‐free survival (PFS), and disease‐free interval (DFI) in different autophagy scores (AutS) groups of liver hepatocellular carcinoma (LIHC) patients. (B) Analysis of AutS in normal and tumor tissues of LIHC patients. (C and D) Multiple gene correlation analysis with autophagy signatures in LIHC. EMT signature includes *CDH2*, *VIM*, *SNAI1*, *SNAI2*, *TWIST1*, *MMP2*, *MMP3*, *MMP‐9MMP9*, and*ZEB1*. TPM, transcripts per million. (E) HepG2 and Hep3B cells were treated with EBSS without serum for 24 and 36 h, and protein levels were examined by western blot (WB). (F) HepG2 and Hep3B cells were treated with 20 μM FCCP for 6 h. Prior to treatment, the cells were pretreated with 10 nM BafA1 for 1 h. The protein levels were detected using WB analysis. The results were analyzed and normalized against the expression levels at 0 h. (G) Analysis of STIM1 expression in normal and tumor tissues of LIHC patients. RSEM, RNA‐Seq by expectation‐maximization. (H) Correlation analysis between *STIM1* and autophagy or EMT related genes. Data of panels (A–D) and (G and H) were obtained from TCGA. Data of panels (E and F) are expressed as mean ± SEM (*n* = 3).

Autophagy has been reported to be involved in multiple stages of cancer, particularly tumorigenesis and metastasis.^23^, ^24^ In order to investigate the correlation between autophagy and HCC progression, we conducted Gene Set Variation Analysis (GSVA) using an autophagy‐related gene set (*MAP1LC3B, ATG10, ATG12, ATG13, ATG3, ULK1*) obtained from The Cancer Genome Atlas (TCGA) HCC patient database. The HCC patients were divided into a high autophagy score (AutS) group and a low AutS group based on the mean AutS. Patients with high AutS exhibited better overall survival (OS), longer progression‐free survival and disease‐free interval (Figure 1A). Furthermore, AutS was significantly higher in HCC tissues compared to normal para‐cancerous tissues (Figure 1B). Using bioinformatics, we analyzed the correlation between cancer‐related pathways and autophagy in HCC. Our findings revealed that EMT was the most closely associated pathway with autophagy (Figure 1C). Additionally, we observed a significantly positive correlation between the EMT score and AutS in HCC patients from the TCGA database (Figure 1D). Furthermore, we conducted experiments where autophagy was induced by serum‐free starvation using Earle's Balanced Salt Solution (EBSS). In this experiment, EBSS‐triggered autophagy facilitated EMT by increasing N‐cadherin expression and decreasing E‐cadherin expression in HCC cells after 24 and 36 h (Figure 1E). These results strongly suggest that autophagy plays a significant role in HCC progression, particularly in relation to EMT.

In our previous research, we discovered that STIM1 plays a role in regulating the metastasis of HCC cells as a metabolic checkpoint. Interestingly, we observed that the expression of STIM1 is upregulated during HCC proliferation in a hypoxic microenvironment, but downregulated when EMT begins.^21^ Additionally, we found that after inducing autophagy, the expression of STIM1 increased at 24 h but decreased at 36 h (Figure 1E). Furthermore, STIM1 expression was decreased during autophagy and the EMT process induced by carbonyl cyanide 4‐(trifluoromethoxy) phenylhydrazone (FCCP), but was restored in the presence of the autophagy inhibitor Bafilomycin A1 (BafA1) (Figure 1F). These findings suggest that STIM1 may be involved in autophagic degradation during autophagy‐triggered EMT in HCC cells. In addition, our findings indicate that STIM1 is significantly upregulated in tumor tissue compared to normal tissue in HCC patients (Figure 1G), which is consistent with our previous study.^20^ Furthermore, correlation analysis demonstrated a positive association between STIM1 and autophagy‐related genes as well as EMT‐related genes in the database of HCC patients (Figure 1H). Consistently, patients with both lower STIM1 and AutS have better OS (Figure S1), indicating that the combined inhibition of STIM1 and autophagy may benefit survival of HCC patients. These results provide evidence that STIM1 is temporally regulated during autophagy‐induced EMT in HCC cells.

### 2.2 STIM1 mediates autophagy and EMT of HCC cells

**FIGURE 2:**
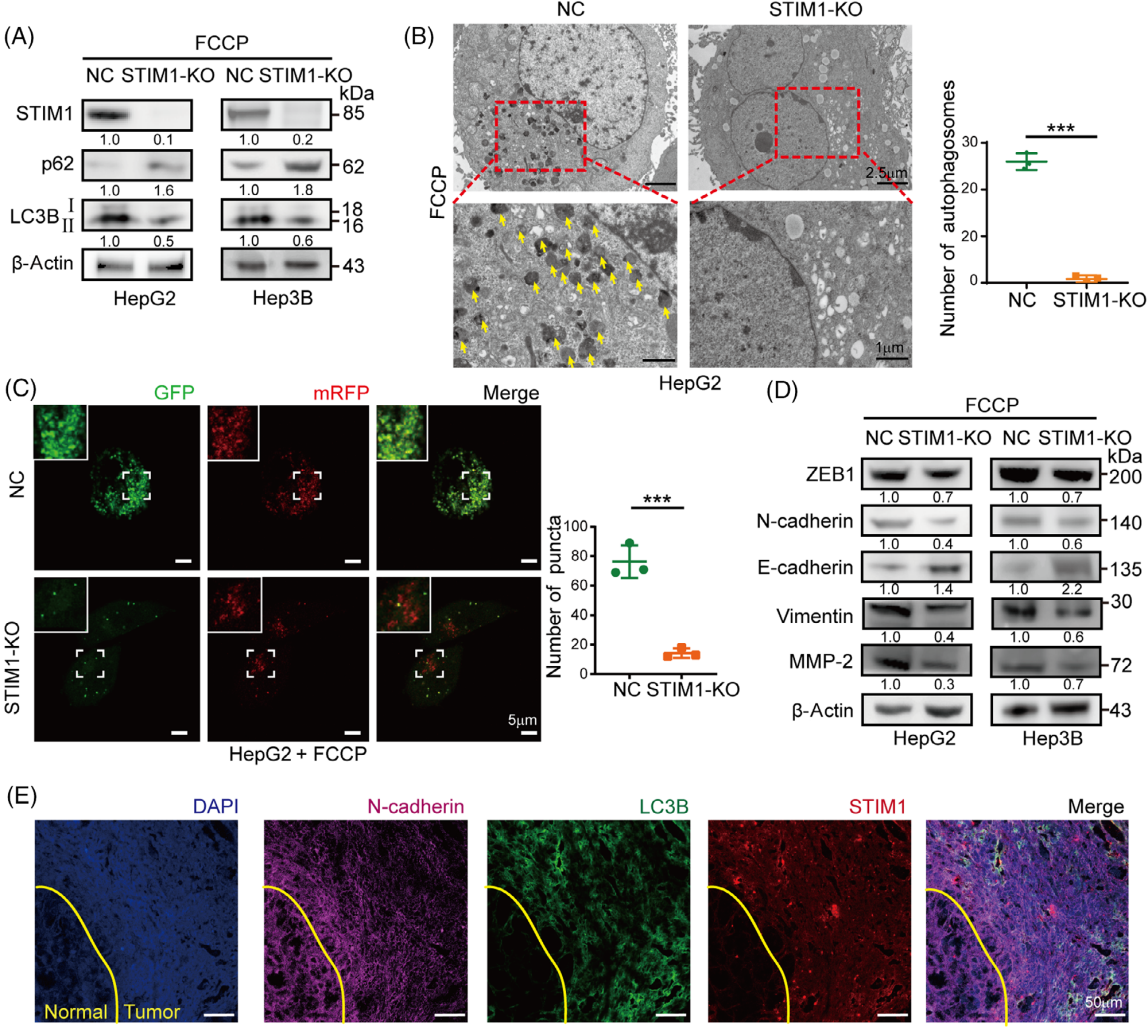
Deficiency of STIM1 reduces autophagy in HCC. (A) After treatment with FCCP (20 μM, 6 h), the autophagy related proteins level of STIM1‐knockout (KO) cells and negative control group (NC) were detected by WB. The results were analyzed and normalized against respective expression in NC cells. (B) After treatment with FCCP (20 μM, 6 h), autophagosomes in NC and STIM1‐KO HepG2 cells, indicated by yellow arrows, were observed by transmission electron microscope (TEM). (C) mRFP‐GFP‐LC3 lentivirus was introduced into NC and STIM1‐KO HepG2 cells. After treatment with FCCP (20 μM, 6 h), yellow puncta represented the autophagosomes. Quantitative analysis of the yellow puncta. (D) After treatment with FCCP (20 μM, 6 h), the EMT proteins level of STIM1‐KO‐ and NC‐HepG2 or Hep3B cells were detected by WB. The results were analyzed and normalized against respective expression in NC cells. (E) The representative image of cancerous peripheral tissue in clinical HCC samples. N‐cadherin (purple), STIM1 (red), and LC3B (green) expression levels were detected by immunofluorescence (IF). The yellow line represents the edge of HCC tissue. The adjacent para‐cancerous tissue is at the bottom left corner (normal) and the HCC tissue (tumor) is at the top right corner. Blue represents DAPI. Data are expressed as mean ± SEM (*n* = 3). ***p < 0.001.

To investigate the role of STIM1 in autophagy and EMT, HepG2 and Hep3B cells, which have high levels of STIM1 protein, were selected to generate STIM1 knockout (KO) cells using the CRISPR/Cas9 system (Figures 2A and S2). Following induction of autophagy by FCCP, a significant decrease in the LC3BII/I ratio and an increase in sequestosome 1 (SQSTM1/p62) expression in the STIM1‐KO cells were observed, indicating a reduction in autophagy levels (Figure 2A). Additionally, electron microscopy analysis revealed a marked decrease in the number of autophagosomes in HepG2 cells upon STIM1 depletion (Figure 2B). Next, mRFP‐GFP‐LC3 lentiviral was transfected into HCC cells to assess autophagic flux. The STIM1‐KO cells exhibited a reduced number of LC3 puncta, indicating a decrease in autophagic flux after FCCP treatment (Figure 2C). Consistent with previous studies,^21^ the STIM1‐KO cells displayed lower EMT features compared to the control groups (Figure 2D), as well as a decreased migration rate and invasion capacity (Figures S3A–C). Furthermore, a higher coexpression of STIM1 and LC3B in the metastasis edge of HCC was observed than that of adjacent normal tissue (Figure 2E). These findings highlight the critical role of STIM1 in both autophagy and EMT processes in HCC cells.

### 2.3 STIM1 regulates autophagy in both SOCE‐dependent and independent manners

**FIGURE 3:**
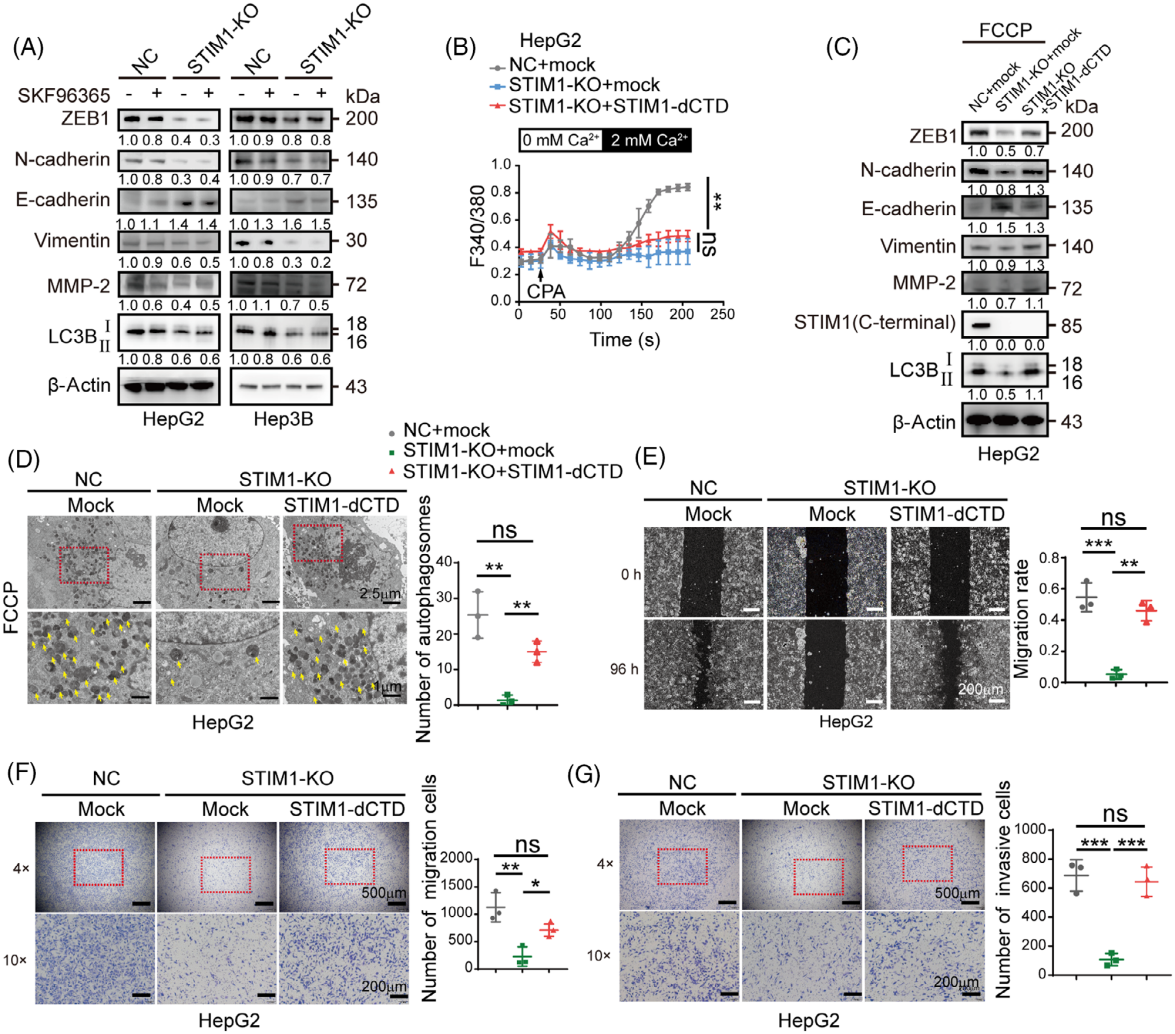
STIM1 mediates autophagy in both SOCE‐dependent and independent manners. (A) NC and STIM1‐KO cells with or without pretreatment of SKF96365 (10 μM, 24 h) were disposed with FCCP (20 μM, 6 h), protein levels were examined by WB. (B) Ca^2+^ mobilization upon cyclopiazonic acid (CPA 20 μM) challenge in HepG2 cells, STIM1‐KO, STIM1‐KO+STIM1‐dCTD cells, respectively. Data are expressed as mean ± SEM (*n* = 5). (C) After treatment with FCCP (20 μM, 6 h), the protein levels in these cells were detected by WB. The STIM1 antibody here specifically recognizes the C‐terminal of STIM1. The results were analyzed and normalized against expression in NC cells. (D) After treatment with FCCP (20 μM, 6 h), autophagosomes, indicated by yellow arrows, were observed under TEM. (E–G) The metastatic capacity of NC, STIM1‐KO, and STIM1‐KO+STIM1‐dCTD HepG2 cells was examined by wound healing assay (E), transwell migration assay (F) and transwell invasion assay (G). The number of migration cells or invasive cells was counted in 10× field of images (F and G). Data of (A, C–G) are expressed as mean ± SEM (*n* = 3). *p < 0.05, **p < 0.01, ***p < 0.001, ns represents no significant difference.

We then speculated whether SOCE is solely responsible for the regulation of autophagy by STIM1. In contrast to STIM1‐KO, the SOCE inhibitor SKF96365 only showed a minimal impact on reducing the autophagy level induced by FCCP in HCC cells (Figure 3A). This implies that there is an alternative mechanism, independent of SOCE but involving STIM1, that is necessary for autophagy. To further investigate the underlying mechanism, we generated a lentivirus containing a functional mutant of STIM1 called STIM1‐dCTD. This mutant lacks SOCE activation activity because it has a deleted C‐terminal domain (aa441‐685). We then introduced STIM1‐dCTD into the STIM1‐KO cells (Figure 3B). Interestingly, the overexpression of STIM1‐dCTD significantly enhanced the formation of autophagosomes, and increased the ratio of LC3BII/I in STIM1‐KO HepG2 cells (Figures 3C and D). In addition, the overexpression of STIM1‐dCTD in cells significantly augmented the EMT features. This was characterized by increased expressions of zinc‐finger‐enhancer protein 1 (ZEB1) and N‐cadherin, decreased expression of E‐cadherin, and enhanced migration and invasion capabilities (Figures 3E–G). These findings suggest that STIM1 plays a critical role in regulating autophagy through both SOCE‐dependent and independent mechanisms in HCC cells.

### 2.4 STIM1 directly interacts with LC3B through SAM domain

**FIGURE 4:**
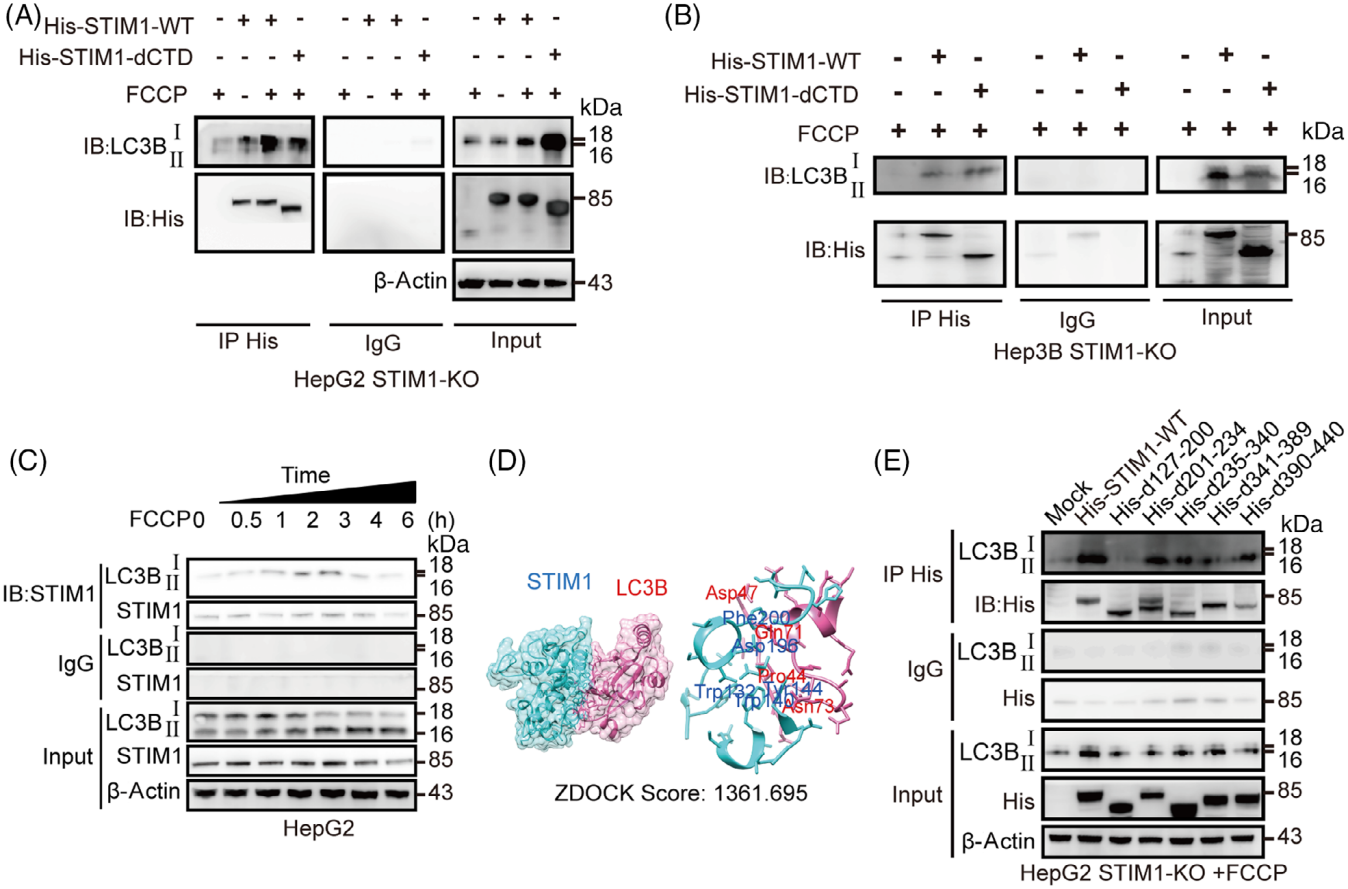
STIM1 directly binds with LC3B through SAM domain. (A and B) STIM1‐KO HepG2 cells (A) and Hep3B cells (B) were transfected with His‐STIM1‐widetype (WT), His‐STIM1‐dCTD, subsequently treated with FCCP (20 μM, 6 h). Cell extracts were immunoprecipitated with His antibody and immunoblotted with antibodies against LC3B. IP, immunoprecipitation; IB, immunoblotting. (C) HepG2 cells were treated by FCCP (20 μM) with different time intervals (0, 0.5, 1, 2, 3, 4, 6 h). Cell extracts were immunoprecipitated with anti‐STIM1 antibody and immunoblotted with antibodies against LC3B. (D) Three‐dimensional (3D) chart and binding model of STIM1 with LC3B were predicted by using the ZDOCK. (E) STIM1‐KO cells were transfected with His‐tagged STIM1‐WT or STIM1 variants with different domain depletions. Following transfection, the cells were treated with FCCP (20 μM, 6 h). Cell extracts were then subjected to immunoprecipitation using His antibody and immunoblotted with antibodies against LC3B. Data of panels (A–C and E) are expressed as mean ± SEM (n = 3).

In light of STIM1 being an ER structural protein that is located in the same area as autophagy initiation,^8^ we next investigated whether STIM1 facilitates autophagy by interacting with autophagy‐related proteins. LC3B is a crucial autophagic ubiquitin‐like protein involved in the formation of autophagosomes. We observed a strong interaction between STIM1 and LC3B after FCCP treatment (Figures 4A and B), suggesting that STIM1 may influence autophagosome formation mediated by LC3B. Furthermore, this interaction between STIM1 and LC3B gradually increased within 3 h after FCCP treatment, which was consistent with the LC3BII/I ratio. However, between 3 and 6 h after FCCP administration, the combination of STIM1 and LC3B gradually decreased (Figure 4C). These findings indicate that STIM1 binds with LC3B during the process of autophagosome formation and is subsequently degraded in autophagosomes or autolysosomes in HCC cells.

After establishing docking models to predict the binding region of STIM1 and LC3B, we identified five possible binding sites located on the SAM domain of STIM1 (Figure 4D). This prediction was supported by the observation that STIM1‐dCTD could also form a complex with LC3B (Figure 4A). To further investigate their interaction domains, we performed coimmunoprecipitation experiments using lysates of STIM1‐KO HCC cells overexpressing full‐length STIM1 or five different deletion mutations of STIM1 along with full‐length LC3B. Our results showed that the absence of the SAM domain (d127‐200) resulted in a decreased ratio of LC3BII/I, which significantly reduced the interaction between STIM1 and LC3B (Figure 4E). These data suggest that STIM1 directly interacts with LC3B through SAM domain during autophagy process.

### 2.5 Deletion of SAM domain in STIM1 diminishes its autophagy‐promoting ability in HCC cells

**FIGURE 5:**
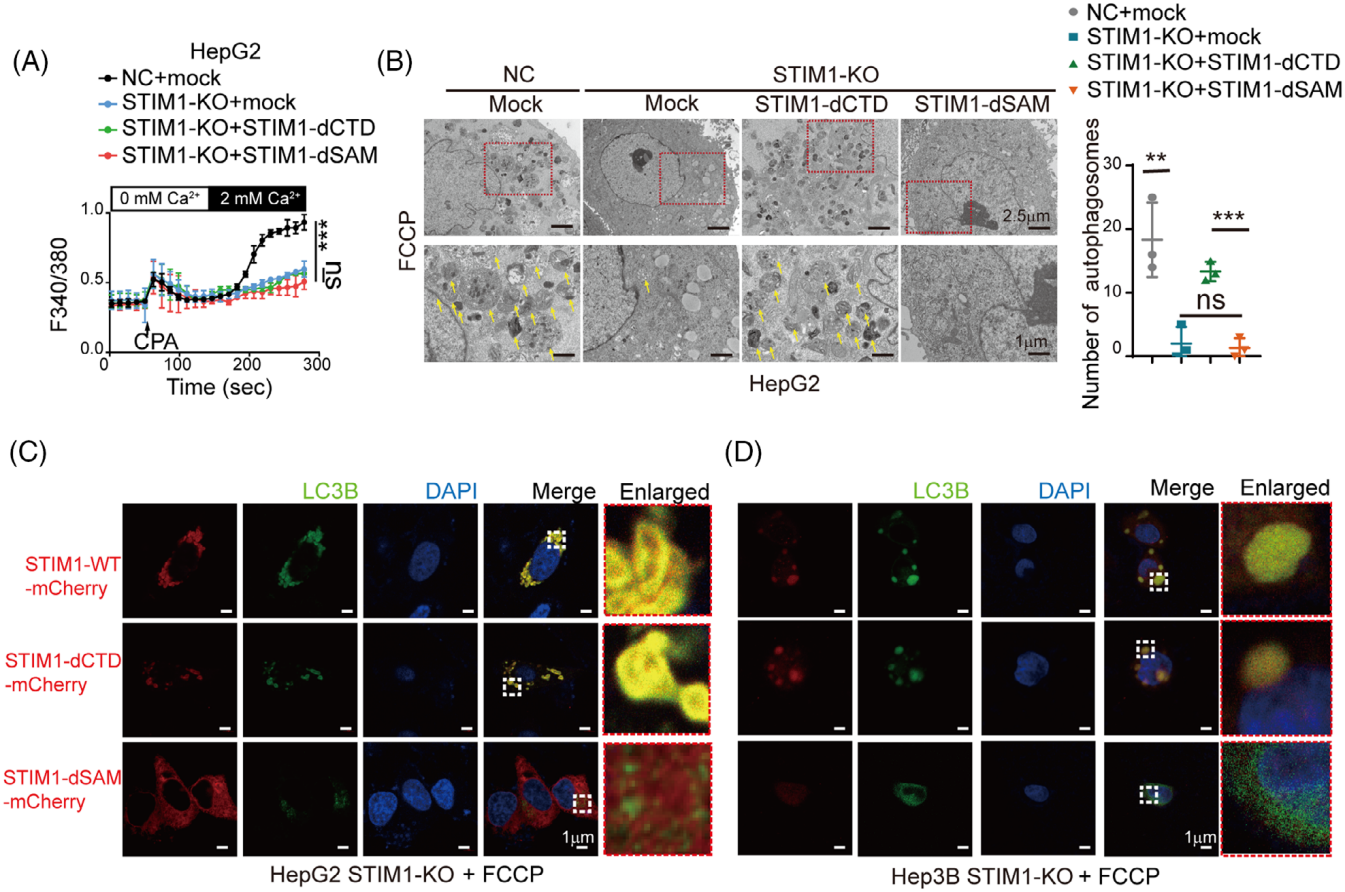
STIM1 facilitates autophagy through interacting with LC3B through its SAM domain. (A) Ca^2+^ mobilization upon CPA (20 μM) challenge in NC, STIM1‐KO, STIM1‐KO+STIM1‐dCTD and STIM1‐KO+STIM1‐dSAM HepG2 cells, respectively; data are expressed as mean ± SEM (*n* = 5). (B) After treatment with FCCP (20 μM, 6 h), autophagosomes were observed by TEM, and autophagosomes were indicated by yellow arrows. (C and D) STIM1‐KO HepG2 cells (C) and Hep3B cells (D) were introduced with mCherry‐tagged STIM1‐WT, mCherry‐tagged STIM1‐dCTD, mCherry‐tagged STIM1‐dSAM. The cells were treated with FCCP (20 μM, 6 h) and then subjected to immunofluorescence staining for LC3B (green) and DAPI (blue). The amplified images of the selected regions (white) are shown on the right. Data of panels (B–D) are expressed as mean ± SEM (*n* = 3). **p < 0.01, ***p < 0.001, ns represents no significant difference.

The SAM domain is known to be essential for the oligomerization of STIM1, which is required for the activation of Orai1.^16^ To further investigate the role of the SAM domain of STIM1 in autophagy and EMT, we transfected lentivirus with the STIM1 variant of deletion SAM domain (STIM1‐dSAM) into STIM1‐KO HCC cells. Similar to STIM1‐dCTD, STIM1‐dSAM also failed to trigger SOCE in STIM1‐KO cells (Figure 5A). However, the introduction of STIM1‐dSAM resulted in a lower level of autophagosomes compared to STIM1‐dCTD (Figure 5B). Additionally, cells expressing STIM1‐dCTD exhibited a higher number of LC3B puncta compared to cells expressing STIM1‐dSAM, indicating the crucial role of the SAM domain of STIM1 in inducing autophagy (Figure 5B). Moreover, immunofluorescence staining revealed that STIM1‐dCTD colocalized more with LC3B in the cytosol, while STIM1‐dSAM did not show any colocalization with LC3B in HCC cells. This further supports the significance of the SAM domain in mediating protein‐binding between STIM1 and LC3B (Figures 5C and D). Taken together, these findings suggest that STIM1 forms a complex with LC3B through its SAM domain, promoting the formation of autophagosomes and enhancing autophagy in HCC cells.

### 2.6 STIM1/LC3B complex contributes to HCC metastasis through activating autophagy

**FIGURE 6:**
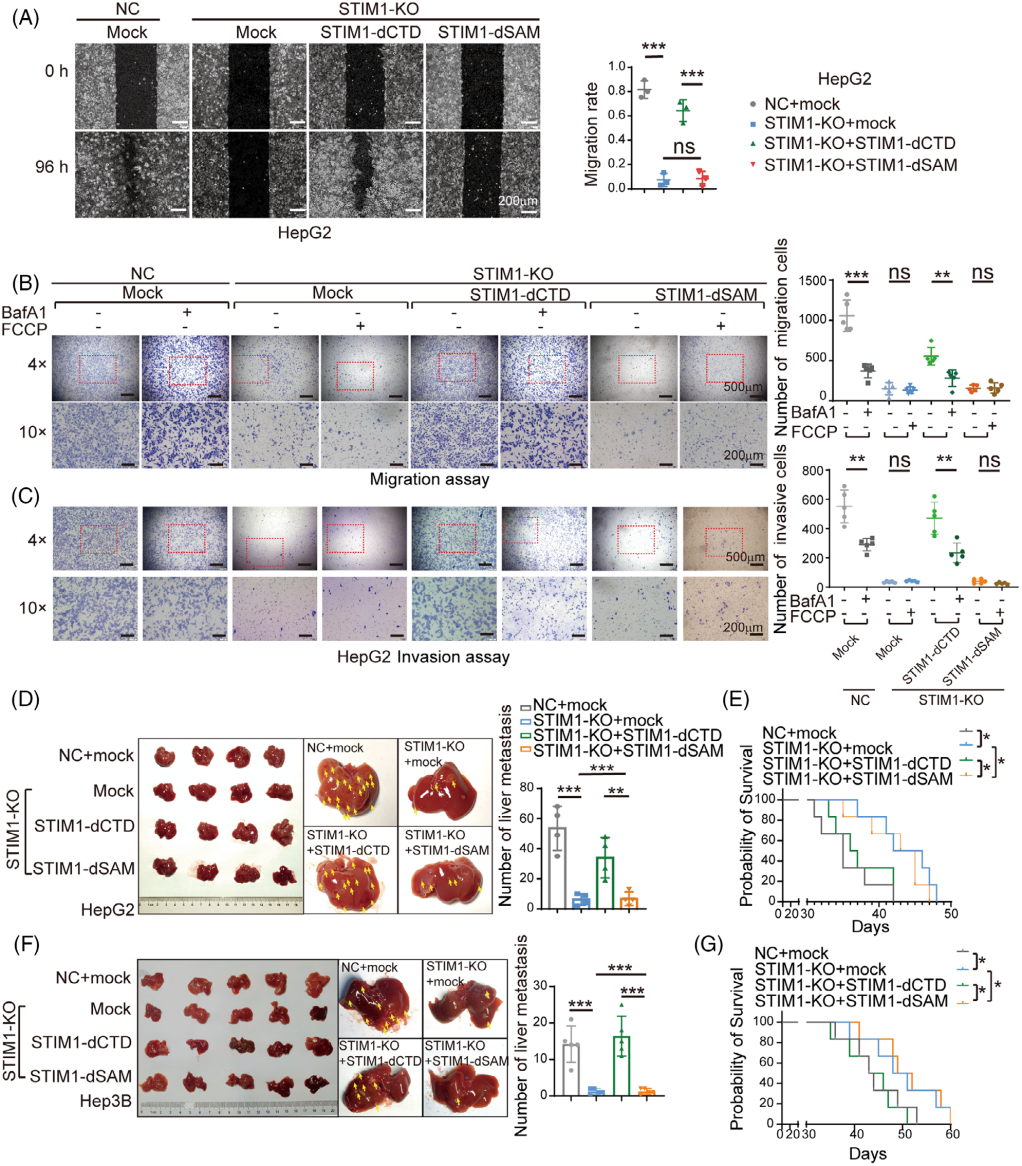
STIM1/LC3B complex accelerated HCC metastasis in vitro and in vivo. (A) Migratory capabilities of HCC cells were examined by wound healing assay; *n* = 3. (B and C) The influence of BafA1 (10 nM) or FCCP (20 μM) on migration capabilities was determined by transwell migration (B) and invasion assay (C). The number of migration cells was counted in 10× field of images; *n* = 5. (D) Metastatic capabilities were evaluated in the intrahepatic metastasis model and metastasis lesions on the liver surface were pointed out by yellow arrows (*n* = 4 mice per group). (E) Mice survival rate was evaluated in the intrahepatic metastasis model (*n* = 5 mice per group). (F) Metastatic capabilities were evaluated by the splenic vein injection metastasis model and metastasis lesions on the liver surface were pointed out by yellow arrows (*n* = 5 mice per group). (G) Mice survival rate was evaluated in the splenic vein injection metastasis model (*n* = 6 mice per group). Data are expressed as mean ± SEM, *p < 0.05, **p < 0.01, ***p < 0.001, ns represents no significant difference.

Given the significant role of autophagy in HCC metastasis, we proceeded to investigate the involvement of the STIM1/LC3 complex in this process. We found that STIM1‐dCTD, rather than STIM1‐dSAM, notably enhanced the migration ability of HepG2 STIM1‐KO cells (Figure 6A). To further investigate the role of autophagy in STIM1 modulation of EMT, BafA1 was used to block autophagy. In the presence of BafA1, the migration and invasion capability enhanced by STIM1‐dCTD was significantly reduced in STIM1‐KO HepG2 cells. Additionally, FCCP failed to induce autophagy and accelerate the migration and invasion rate in STIM1‐dSAM expressing STIM1‐KO HepG2 cells (Figures 6B and C). These findings suggest that the SAM domain of STIM1 plays a predominant role in promoting autophagy and EMT in HCC cells.

Next, we examined the impact of STIM1 on the metastasis of HCC cells in vivo. Using an orthotopic liver injection model, we observed that the absence of STIM1 significantly reduced the metastatic ability of HepG2 cells. Interestingly, after ectopically expressing STIM1‐dCTD (but not STIM1‐dSAM), a dramatic enhancement in intrahepatic metastasis of STIM1‐KO cells was observed (Figure 6D). Furthermore, mouse models injected with STIM1‐KO cells or ectopically expressing STIM1‐dSAM cells exhibited a significantly improved survival rate (Figure 6E). We also utilized a splenic vein injection model and obtained consistent results (Figures 6F and G). Compared with NC groups, STIM1‐KO led to significantly decreased LC3B level in metastatic lesions. Introduction of STIM1‐dCTD, other than STIM1‐dSAM, possesses the capability of restoring LC3B expression (Figure S4). Collectively, our findings suggest that STIM1 promotes autophagy by interacting with LC3B through its SAM domain, forming a complex that leads to EMT and metastasis in HCC cells.

## 3 DISCUSSION

Abundant evidence suggests that autophagy facilitates EMT and tumor metastasis in the late stages of tumor development.^5^, ^6^ In this study, we report that STIM1 is temporally regulated during autophagy‐triggered EMT in HCC cells. Furthermore, we demonstrate that STIM1 promotes autophagy through both SOCE‐dependent and SOCE‐independent mechanisms. Mechanistically, STIM1 interacts with LC3B through its SAM domain, thereby promoting the formation of autophagosomes via a SOCE‐independent pathway. This novel SOCE‐independent function of STIM1 in regulating autophagy has implications for HCC metastasis.

As the primary mediator of SOCE, STIM1 multimers interact with Orai1 at the junction between the ER and the plasma membrane (PM) through the STIM1‐Orai1 activating region located at the C‐terminus of STIM1, following depletion of ER calcium.^17^, ^18^ Additionally, STIM1 interacts directly with the stimulator of interferon genes to keep it in an inactive state on the ER membrane, resulting in a reduced type I interferon response.^25^ Previous studies have demonstrated that STIM1‐mediated SOCE regulates hypoxia‐driven hepatocarcinogenesis, metastasis, and self‐renewal of cancer stem cells in HCC.^20^, ^21^, ^26^ It is widely accepted that the cancer‐promoting function of STIM1 is dependent on SOCE.^27^ However, in this study, we provide evidence that STIM1 also facilitates autophagy flux through direct binding with LC3B, which is independent of SOCE. These findings reveal a novel non‐SOCE function of STIM1.

During the process of autophagy, proteins and other components that require degradation are enclosed within autophagosomes.^28^ In this study, it was observed that STIM1 expression was upregulated within the first 24 h of autophagy induced by starvation, but attenuated after 36 h. Additionally, the presence of an autophagy inhibitor restored STIM1 expression. These findings suggest that STIM1 undergoes degradation during the later stages of autophagy. Interestingly, the behavior of STIM1 in the autophagy process mirrors its behavior in metastasis. In tumor growth, STIM1 stabilizes the Snail1 protein by activating the CaMKII/AKT/glycogen synthase kinase 3 beta pathway. Consequently, the increased levels of Snail1 suppress STIM1 upon the onset of EMT.^21^ These results indicate that STIM1 plays a crucial role in the temporal and differential regulation of autophagy and metastasis in HCC cells.

There are still some limitations in our present study that require further investigation. First, it is unclear how the SAM domain of STIM1, which is typically located in the ER lumina, translocates to interact with LC3B in the autophagosome. Some research suggests that during autophagosome formation, the ER membrane can be engulfed as the inner membrane,^29^ potentially allowing STIM1 to interact with LC3B in the autophagosome lumen. Second, docking model predictions indicate that two benzene ring structures (Tyr144, Phe200) in the SAM domain of STIM1 have the highest affinity with LC3B, suggesting that these amino acid sites may be crucial for their binding.

In conclusion, our findings demonstrate that STIM1 plays an important role in facilitating autophagy and EMT by directly interacting with LC3B. Additionally, we discover a novel function of STIM1 in autophagy that is independent of SOCE. These results suggest that targeting the SAM domain of STIM1 could be a promising therapeutic strategy for preventing metastasis in HCC.

## 4 MATERIALS AND METHODS

### 4.1 Human HCC tissue specimens

Human HCC tissue specimens were obtained from the Chongqing University Cancer Hospital. The use of clinical specimens in this study was approved by Ethical Review Board of the Chongqing University Cancer Hospital ethics committee, and informed written consent was obtained from all participants.

### 4.2 Cell lines

HepG2, HuH‐7, MHCC97‐H, HCCLM3, BEL‐7402, PLC/PRF/5, SMMC‐7721, and Hep3B were purchased from the American Type Culture Collection (Rockville, MD, USA). All cell lines used in this study were authenticated and tested for mycoplasma. Passages with a value of less than 10 were utilized.

### 4.3 Animals

All male BALB/c nude mice were purchased from Vital River (Beijing, China). For orthotopic tumor models, 5×10^4^ cells were resuspended in 50 μL of PBS and injected into the left lateral lobe of the liver. After a period of 4 weeks, the mice were sacrificed in order to observe the metastasis. In the case of splenic vein injection tumor models, 1×10^7^ cells were resuspended in 200 μL of PBS and injected into the splenic vein. After a period of 6 weeks, the mice were sacrificed and the metastasis lesions were counted by dissecting the liver. All experimental protocols were approved by the Army Medical University Animal Ethics Committee and strictly adhered to the guidelines of the National Institutes of Health regarding animal welfare.

### 4.4 Chemicals and reagents

SKF96365 (HY‐100001), puromycin (HY‐B1743), BafA1 (HY‐100558), cyclopiazonic acid (CPA; HY‐N6771), and FCCP (HY‐100410) were obtained from MedChemExpres (Monmouth Junction, NJ, USA). EBSS (14155063), Fura2‐AM (F1221), and Lipofectamine 3000 (L3000015) were obtained from Thermo Fisher Scientific (Waltham, MA, USA). Dimethysulfoxide (34869) and CPA (239805) were purchased from Sigma–Aldrich (St. Louis, MO, USA).

### 4.5 Plasmids and lentiviral

To generate truncated fragments of STIM1 with different deleted domains, including d127‐200aa (deletion of SAM domain), d201‐234aa (deletion of TM domain), d235‐340aa (deletion of CC1 domain), d341‐389aa (deletion of CC2 domain), and 390−440aa (deletion of CC3 domain), a His‐tag was added to the C‐terminus of each fragment. These fragments were then subcloned into the pCMV3‐C‐His vector (Genscript, Wuhan, China). Lentivirus expressing sgRNAs for deleting STIM1 and lentivirus over‐expressing the STIM1‐dCTD mutant have been previously described.^21^ Lentivirus carrying the STIM1‐dSAM expression element (lacking 127−200 aa) and mRFP‐GFP‐LC3 lentivirus were obtained from Genechem (Shanghai, China). The procedures for lentivirus infection, plasmid transfection, and selection of stable expressing STIM1‐mutant cells were previously described.^21^

### 4.6 Western blotting and coimmunoprecipitation

LC3B antibody (3868S), N‐cadherin antibody (13116S), E‐cadherin antibody (3195S), STIM1 antibody (5668S), SQSTM1/p62 antibody (23214S), ZEB antibody (83243SF), MMP‐2 antibody (40994S), Vimentin antibody (5741S), and His‐tag antibody (12698S) were purchased from Cell Signaling Technology (Danvers, MA, USA). β‐Actin antibody (81115‐1‐RR) was purchased from Proteintech (Wuhan, China). The western blot was performed according to previously described methods.^20^ For coimmunoprecipitation, cells were seeded and grown on 10 cm dishes overnight. After treatment, cells were harvested in lysis buffer with PMSF. The supernatants collected from centrifugation were immunoprecipitated with His‐tag antibody at 4°C overnight. Then, the mixture was blended with protein A/G PLUS‐agarose and incubated at 4°C for 2 h. Next, the mixture was washed by centrifuging five times with cold lysis buffer containing PMSF and the sediment was heated for denaturation with SDS buffer. Finally, immunoblotting analysis was performed.

### 4.7 Observation of autophagosomes by transmission electron microscope

Cell resuspension solution was centrifuged at 200 g. The sediment was immediately fixed with 2.5% glutaraldehyde in 0.1 mol/L sodium cacodylate and stored at 4°C until embedding. Afterwards, samples were postfixed with 1% osmium tetroxide and dehydrated using an increasing concentration gradient of ethanol and propylene oxide. Subsequently, the samples were embedded and ultrathin (50–60 nm) sections were cut using an ultramicrotome. Finally, the images were examined using a JEM‐1200 electron microscope (JEOL, Japan) at 80 kV after staining with 3% uranyl acetate and lead citrate.

### 4.8 Calcium imaging

Calcium imaging was performed following the established protocol.^21^ Briefly, cells were seeded on circular glass slides coated with poly‐d‐lysine (Nest, Wuxi, China) and incubated overnight. Fura2‐AM was used to label intracellular Ca^2+^, and the fluorescence signal was measured using an inverted fluorescence microscope (Nikon, Tokyo, Japan). The Ca^2+^ signal was collected at wavelengths of 340 and 380 nm. To investigate the impact of different solutions, we replaced the initial solution with either HBSS containing CPA or HBSS containing 2 mM CaCl_2_. The fluorescence signals at 340 and 380 nm were recorded and the ratios were calculated.

### 4.9 Immunofluorescence

Cells were seeded on circular glass coated with poly‐d‐lysine and cultured overnight. Afterward, cells were fixed using 4% Polyoxymethylene and washed three times. Subsequently, cells were blocked with BSA and incubated with the primary antibody overnight at 4°C. Following this, the glass was washed and incubated with the second antibody for 2 h. Finally, the slides were covered with antifade mounting medium containing DAPI and observed under a ZEISS LSM 880 confocal microscope (ZEISS, Germany).

### 4.10 Docking prediction

The 3D structure of the STIM1/LC3B interaction was constructed using the SWISS‐MODEL server (http://swissmodel.expasy.org/). The structure of STIM1 (PDB ID: 2K60) and LC3B (PDB ID: 2ZJD 1−125) was utilized. Molecular docking calculations were performed using the ZDOCK 3.0/3.0.2 Scoring Function.^30^

### 4.11 Cell invasion and migration assay

The wound‐healing assay, transwell assay, and immunofluorescence were performed as previously described.^21^

### 4.12 Quantification and statistical analysis

GSVA analysis was conducted by using the GSCA website (http://bioinfo.life.hust.edu.cn/GSCA) with data obtained from the TCGA database. Correlation analysis was performed by using the GEPIA website (http://gepia.cancer‐pku.cn/) with data also obtained from the TCGA database. Statistical analysis was carried out using GraphPad Prism7 (version 7; La Jolla, CA). All data were presented as mean ± SEM and analyzed using Student's *t*‐test or one‐way ANOVA. Survival analysis was analyzed using Mantel–Cox test. Statistical significance was defined as *p* values < 0.05.

## AUTHOR CONTRIBUTIONS

H. Z., Y. L., and S. Y. designed the research; J. W., Y. Z., Y. X., Y. C., and R. R. performed experimental work; L. W. performed the docking prediction; J. W. and Q. X. analyzed the data; Y. L., J. W., and H. Z. wrote the manuscript. All authors have read and approved the final manuscript.

## CONFLICT OF INTEREST STATEMENT

The authors declare no conflict of interest.

## ETHICS STATEMENT

The research protocol and written informed consent conformed to the principles of medical ethics. The use of clinical specimens in this study was approved by the Ethical Review Board of the Chongqing University Cancer Hospital ethics committee, and the Ethics Number is CZLS2023137‐A. All animal experimental protocols were approved by the Army Medical University Animal Ethics Committee, and the Animal Ethics Number is AMUWEC20227508. All animal experimental protocols were strictly followed the guidelines of the National Institutes of Health Guidelines regarding animal welfare.

## Supporting information

Supporting Information

## Data Availability

All data supporting the findings of this study are available from the authors upon reasonable request.
