# Supplementary material for: Stromal interaction molecule 1/microtubule‐associated protein 1A/1B‐light chain 3B complex induces metastasis of hepatocellular carcinoma by promoting autophagy
Source: MedComm (2020). 2024 Feb 9;5(2):e482. doi: 10.1002/mco2.482 (PMC10857778; doi:10.1002/mco2.482)
Supplement: Supplementary file 1 — Supporting Information [file MCO2-5-e482-s001.docx]

**Stromal-interaction molecule 1/ microtubule-associated protein 1A/1B-light chain 3B complex induces metastasis of hepatocellular carcinoma by promoting autophagy**

**Running Title:** STIM1/ LC3B complex mediates autophagy

Jingchun Wang^1#^, Qichao Xie^2#^, Lei Wu^3^, Yu Zhou^3^, Yanquan Xu^4^, Yu Chen^3^, Jiangang Zhang^3^, Ran Ren^3^, Shiming Yang^1^*, Yongsheng Li^3^*, Huakan Zhao^3^*

1. Department of Gastroenterology, Second Affiliated Hospital, Army Medical University, Chongqing 400037, China.

2. Department of Oncology, The Third Affiliated Hospital, Chongqing Medical University, Chongqing 401120, China.

3. Department of Medical Oncology, Chongqing University Cancer Hospital, Chongqing 400030, China.

4. Clinical Medicine Research Center, Second Affiliated Hospital, Army Medical University, Chongqing 400037, China.

**^#^** These authors contributed equally to this work.

*** Correspondence** ([yangshiming@tmmu.edu.cn](mailto:yangshiming@tmmu.edu.cn)) (Y.S.), (lys@cqu.edu.cn) (L.Y.) and (ZHKK2011@126.com) (Z.H.)

**Supplemental information**

**Number of Supplemental Figures = 4.**

**
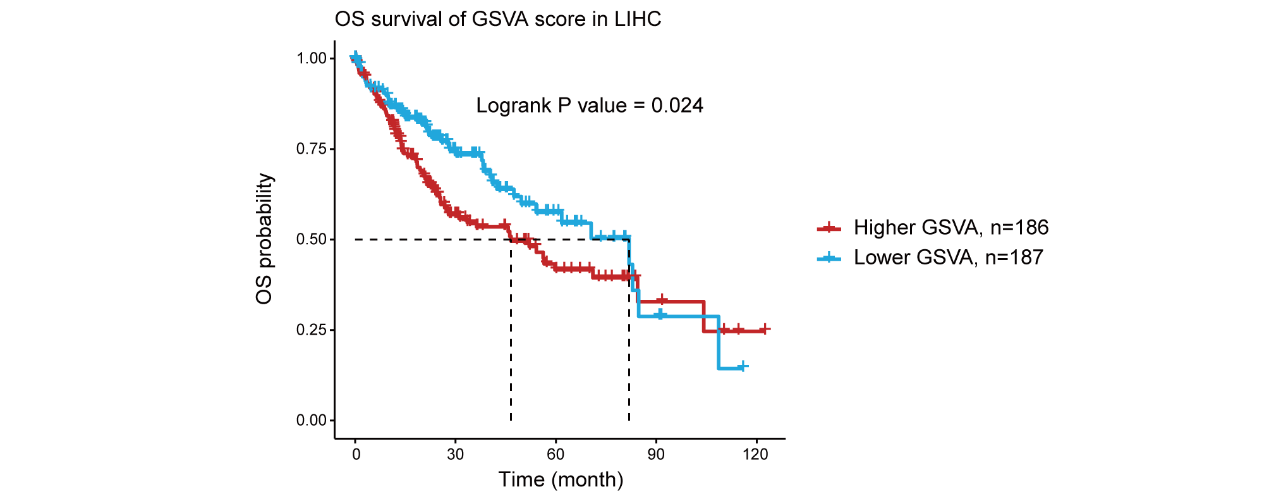
FIGURE S1**

**Figure S1. Overall survival of LIHC patients with different STIM1 and autophagy level.** GSVA analysis was conducted to divided the patients with different levels of STIM1 and autophagy score using the GSCA website (http://bioinfo.life.hust.edu.cn/GSCA) with data obtained from the TCGA database.

**FIGURE S2**


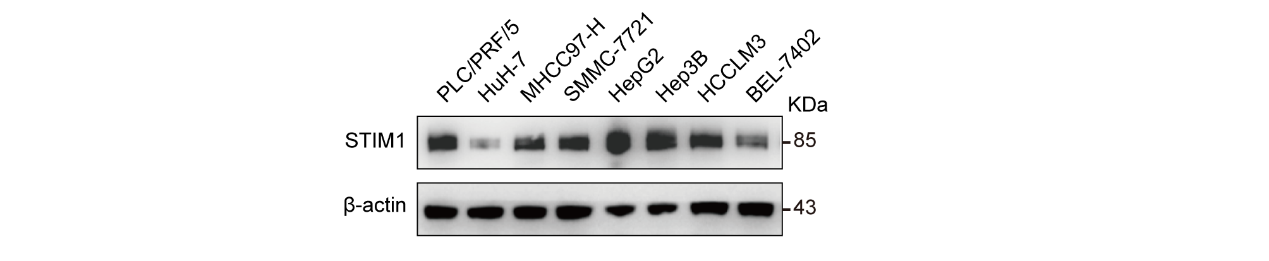


**Figure S2. STIM1 protein expression in HCC cell lines.** STIM1 expression in eight HCC cell lines (PLC/PRF/5, HuH-7, MHCC97-H, SMMC-7721, HepG2, Hep3B, HCCLM3 and BEL-7402) was detected by WB, and β-actin was used as a control of total protein.

**FIGURE S3**


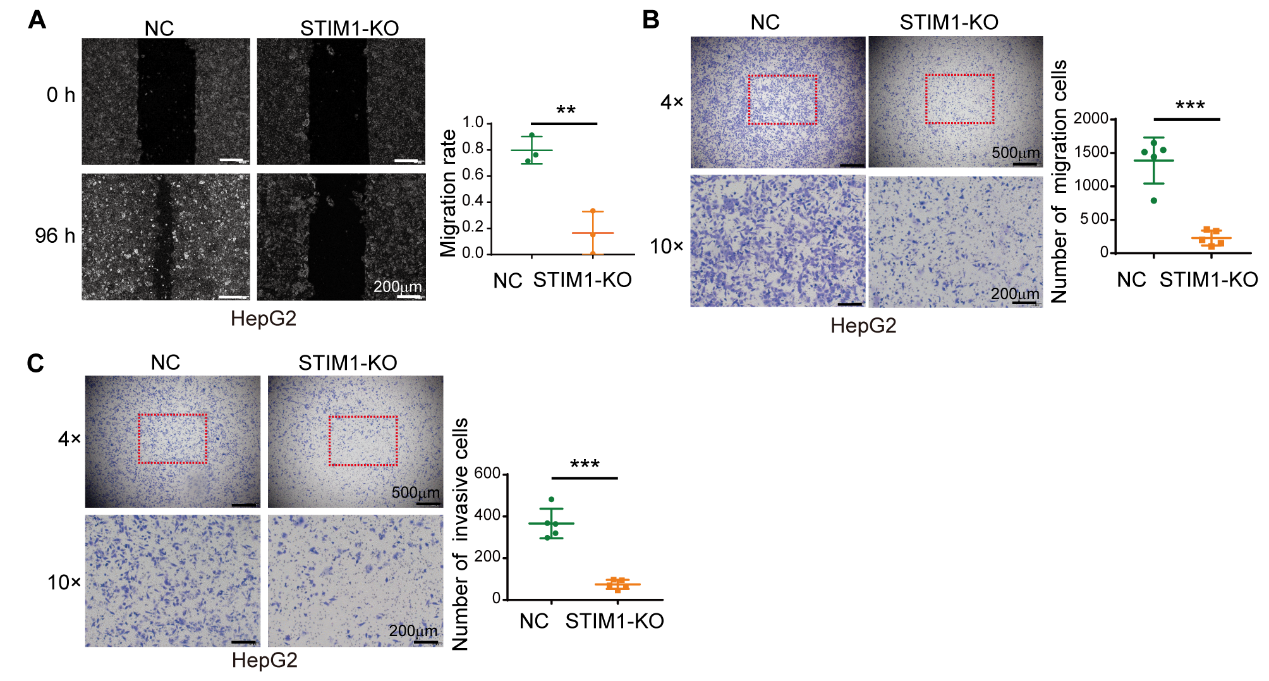


**Figure S3. Knockout of STIM1 dampens EMT in HCC cells.** (**A**-**C**) The metastatic capacity of NC and STIM1-KO HepG2 cells were examined by wound healing assay (**A**), transwell migration assay (**B**) and transwell invasion assay (**C**). Data of **A** are expressed as mean ± SEM (n = 3). Data of **B, C** are expressed as mean ± SEM (n = 5) ***p* < 0.01, ****p* < 0.001.

**FIGURE S4**


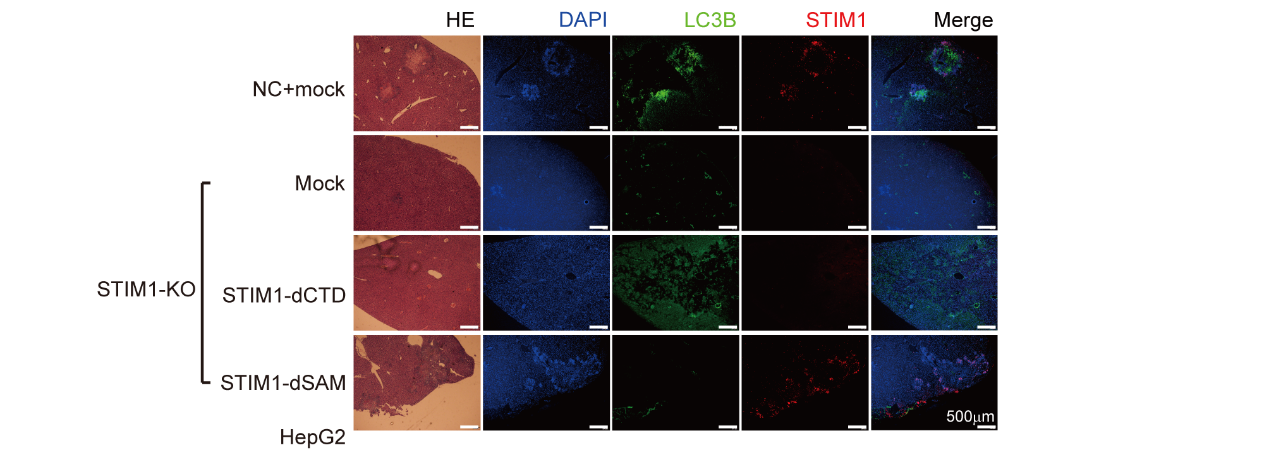


**Figure S4. STIM1/LC3B complex expressions in metastasis lesions.** The representative image of metastasis lesions in intrahepatic metastasis mode was detected by hematoxylin-eosin staining (HE). STIM1 (red) and LC3B (green) protein expressions were detected by IF, Blue represent DAPI, (n = 6 mice per group).
